# Establishment of Glycosaminoglycan Assays for Mucopolysaccharidoses

**DOI:** 10.3390/metabo4030655

**Published:** 2014-08-11

**Authors:** Shunji Tomatsu, Tsutomu Shimada, Robert W. Mason, Adriana M. Montaño, Joan Kelly, William A. LaMarr, Francyne Kubaski, Roberto Giugliani, Aratrik Guha, Eriko Yasuda, William Mackenzie, Seiji Yamaguchi, Yasuyuki Suzuki, Tadao Orii

**Affiliations:** 1 Nemours/Alfred I duPont Hospital for Children, Wilmington, DE 19803, USA; E-Mails: tsutomu.shimada@nemours.org (T.S.); rmason@nemours.org (R.W.M.); fkubaski@udel.edu (F.K.); aratrik@udel.edu (A.G.); Eriko.Yasuda@nemours.org (E.Y.); wmackenz@nemours.org (W.M.); 2 Department of Pediatrics, Saint Louis University, St. Louis, MO 63104, USA; E-Mail: montana@slu.edu; 3 Agilent Technologies, Inc., Wakefield, MA 01880, USA; E-Mails: joan.kelly@agilent.com (J.K.); william.lamarr@agilent.com (W.A.L.); 4 Department of Genetics/UFRGS, Medical Genetics Service/HCPA, Porto Alegre 90035-903, Brazil; E-Mail: rgiugliani@hcpa.ufrgs.br; 5 Department of Pediatrics, Shimane University, Shimane 693-8501, Japan; E-Mail: seijiyam@med.shimane-u.ac.jp; 6 Medical Education Development Center, Gifu University, Gifu 501-1194, Japan; E-Mail: ysuz@gifu-u.ac.jp; 7 Department of Pediatrics, Gifu University, Gifu 501-1194, Japan; E-Mail: orii.tadao@camel.plala.or.jp

**Keywords:** mucopolysaccharidoses, ELISA, tandem mass spectrometry, glycosaminoglycan, chondroitin sulfate, dermatan sulfate, heparan sulfate, keratan sulfate

## Abstract

Mucopolysaccharidoses (MPS) are a group of lysosomal storage disorders caused by deficiency of the lysosomal enzymes essential for catabolism of glycosaminoglycans (GAGs). Accumulation of undegraded GAGs results in dysfunction of multiple organs, resulting in distinct clinical manifestations. A range of methods have been developed to measure specific GAGs in various human samples to investigate diagnosis, prognosis, pathogenesis, GAG interaction with other molecules, and monitoring therapeutic efficacy. We established ELISA, liquid chromatography tandem mass spectrometry (LC-MS/MS), and an automated high-throughput mass spectrometry (HT-MS/MS) system (RapidFire) to identify epitopes (ELISA) or disaccharides (MS/MS) derived from different GAGs (dermatan sulfate, heparan sulfate, keratan sulfate, and/or chondroitin sulfate). These methods have a high sensitivity and specificity in GAG analysis, applicable to the analysis of blood, urine, tissues, and cells. ELISA is feasible, sensitive, and reproducible with the standard equipment. HT-MS/MS yields higher throughput than conventional LC-MS/MS-based methods while the HT-MS/MS system does not have a chromatographic step and cannot distinguish GAGs with identical molecular weights, leading to a limitation of measurements for some specific GAGs. Here we review the advantages and disadvantages of these methods for measuring GAG levels in biological specimens. We also describe an unexpected secondary elevation of keratan sulfate in patients with MPS that is an indirect consequence of disruption of catabolism of other GAGs.

## 1. Introduction

The mucopolysaccharidoses (MPSs) are a group of lysosomal storage diseases (LSDs) caused by deficiency of the lysosomal enzymes required to degrade glycosaminoglycans (GAGs) such as dermatan sulfate (DS), heparan sulfate (HS), keratan sulfate (KS), chondroitin sulfate (CS), and hyaluronan [1,2]. Each type of MPS causes accumulation of particular GAG (s) (Table 1). In MPSs, the undegraded GAGs are stored in lysosomes and extracellular matrix (ECM) of a variety of tissues, secreted into the bloodstream, and excreted in the urine. Accumulated GAGs lead to cell dysfunction and abnormal structure of ECM, causing progressive damage of multiple tissues including CNS, lung, heart, liver, spleen, kidney, joint, and bone. There are 11 known enzyme deficiencies, resulting in seven distinct forms of MPS. The collective incidence is more than 1 in 25,000 live births. Most patients with MPS are asymptomatic as newborns, with subsequent onset of clinical signs and symptoms that include inguinal or umbilical hernia, abnormal development of bones, short stature, coarse hair, hepatosplenomegaly, and mental retardation during infancy or childhood. In most patients, these clinical manifestations progress over several years. While the symptoms and severity of MPS vary with each patient and its subtype of MPS, the average life span in most patients is one to two decades if untreated.

Enzyme replacement therapy (ERT) [3,4,5], hematopoietic stem cell transplantation (HSCT) [6,7,8,9], substrate reduction therapy (SRT) [10,11], gene therapy [12,13], and anti-inflammatory drugs [14,15] are in clinical use or being investigated under clinical trials for patients with some types of MPS. Initiating these treatments at birth or during early stages provides most benefits in the clinical improvement of the disease. Therefore, successful treatment of these disorders depends on early diagnosis. Identification of disease biomarkers is of inevitable importance in diagnosis, clinical severity and its prognosis, pathogenesis, and monitoring for therapies.

**Table 1:** Correlation between mucopolysaccharidoses and GAG (s).

Disorder	Deficient Enzyme	Trait	Chromosome	Primary storage GAG(s)	KS elevation in blood
MPS I (Hurler)	α-L-Iduronidase (IDUA)	AR	4p16.3	DS, HS	↑↑↑
MPS II (Hunter)	Iduronate-2-sulfatase (IDS)	XR	Xq28	DS, HS	↑↑↑
MPS IIIA (Sanfillipo A)	Heparan-N-sulfatase (SGSH)	AR	17q25.3	HS	↑
MPS IIIB (Sanfillipo B)	α-N-Acetylglucoaminidase (NAGLU)	AR	17q21	HS	↑
MPS IIIC (Sanfillipo C)	α-Glucosaminidase acetyltransferase (HGSNAT)	AR	8p11-q13	HS	↑
MPS IIID (Sanfillipo D)	N-Acetylglucosamine 6-sulfatase (GNS)	AR	12q14	HS	NA
MPS IVA (Morquio A)	Galactose 6-sulfatase, N-acetylgalactosamine-6-sulfate sufatase (GALNS)	AR	16q24.3	C6S, KS	↑↑↑
MPS IVB (Morquio B)	β-Galactosidase (GLB1)	AR	3p21.33	KS	↑
MPS VI (Maroteaux-Lamy)	N-Acetylgalactosamine-4-sulfatase (G4S)	AR	5q13.3	C4S, DS	↑↑
MPS VII (Sly)	β-D-Glucuronidase (GUSB)	AR	7q21-q22	C4, 6S, DS, HS	↑↑

AR: autosomal recessive, XR: X-linked recessive, C4S: chondroitin 4-sulfate, C6S: chondroitin 6-sulfate, DS: dermatan sulfate, HS: heparan sulfate, KS: keratan sulfate.

GAGs consist of CS, DS, HS, KS, and hyaluronan. These GAGs are identified in various tissues and cell types, and occupy major components of the extracellular matrix (ECM) and connective tissues. GAGs, except hyaluronan, are sulfated polysaccharides comprising of repeating disaccharides; uronic acid (or galactose) and hexosamines. Polymeric GAGs are covalently attached through a linkage region to core proteins to produce proteoglycans (PGs). PGs are associated with various physiological functions such as hydration and swelling pressure to the tissue to absorb compressional forces, regulation of collagen fibril formation, modification of the activity of transforming growth factor-β, and the major anionic site responsible for the charge selectivity in glomerular filtration. Sulfation patterns in the GAG chains play important roles by permitting interactions, normally of the ionic nature, with growth factors. The core proteins are not just scaffolds for GAGs, containing the domains that have particular biological activities [16]. Many PGs are multifunctional molecules that engage in different specific interactions simultaneously.

Several procedures have been established to measure GAGs. Dye-spectrometric methods including dimethylmethylene blue (DMB) [17,18,19,20,21,22] and alcian blue [23] were developed to measure total urinary GAGs. Thin-layer chromatography (TLC) was used for identification of each specific GAG; however, these methods are not adapted to blood or tissue extracts without prior protease, nuclease or hyaluronidase digestion. Sensitivity and specificity of dye-spectrometric or the TLC method are not sufficient to detect all types of MPS, especially MPS IV. HPLC is a sensitive, reproducible, and accurate method to assay each specific GAG but cannot be applied to mass screening because the method is complex and time-consuming [24,25,26].

ELISA assays for KS and HS in blood and urine were established, indicating a better resolution between normal controls and patients with MPS I, II, III, IVA, and VII, compared with the DMB method [27,28,29]. ELISAs to measure KS, HS, or DS are rapid and reproducible but expensive. Thus, establishment of a simple, accurate, reproducible, and cost-effective GAG assay method is urgently needed to apply to not only clinical indications but also basic research.

We have developed a new approach to assay disaccharides derived from CS, DS, HS, and KS in blood, urine, and/or dried blood spot (DBS) samples by using liquid chromatography tandem mass spectrometry (LC-MS/MS) [30]. The LC-MS/MS method not only shows sensitivity and specificity for detecting all subtypes of MPS, but also monitors therapeutic efficacy in MPS patients and animal models; however, since LC processing is still time consuming, the main drawback of this method could be throughput.

The use of an automated high-throughput mass spectrometry (HT-MS/MS) system (RapidFire) eliminates the chromatographic process, enabling sample-to-sample cycle times to be reduced from minutes to seconds, while maintaining the quality and accuracy of standard LC-MS/MS platform. Each sample is processed within ten seconds, indicating that a single HT-MS/MS system can analyze over one million samples annually. We recently reported that HT-MS/MS could measure HS levels in control human blood much faster than LC-MS/MS, while the HS values produced by HT-MS/MS have a strong correlation with those by LC-MS/MS [30].

During development of highly sensitive ELISA and LC-MS/MS methods, we found that the original concept that “each type of MPS elevates specific GAG(s) in biological specimen based upon the catabolic pathway of GAG” is not always true [28,29,31]. Patients with MPS IVA and IVB have a deficiency of the enzyme that directly involves KS metabolism; N-acetylgalactosamine-6-sulfate sulfatase (GALNS) and β-galactosidase (GLB1), respectively. Therefore, elevation of KS in blood and urine of these types of MPS would be expected; however, it would not be expected that patients with other types of MPS, in which the responsible enzymes do not directly involve the catabolic pathway of KS, have an elevation of KS in blood and urine. In some patients with types of MPS that do not directly involve KS catabolism, levels of KS were as high as that in patients with a severe form of MPS IVA [28,31,32]. The mechanism by which KS is elevated in these types of MPS has not yet been determined.

In this article, we review GAG assays that use ELISA, LC-MS/MS, and HT-MS/MS methods. We also describe a hypothesis to explain the secondary elevation of KS in blood in patients with MPS that do not directly involve metabolic pathways of KS.

## 2. ELISA

### 2.1. Background

Total urinary GAG, a potential biomarker for MPS, is measured spectrometrically by using DMB [17,18,33,34] or Alcian blue [23,35]; however, these methods cannot be applied to blood without protease treatment, since protein in the specimen hinders the binding of the dye to the GAG. Moreover, the dye itself tends to decompose, leading to a high background and false positive results. In particular, levels of total urinary GAGs in a substantial number of patients with MPS IVA are within the normal range, so it is difficult to diagnose this disease based on only urine GAG excretion [18,27,31].

### 2.2. Development of Sandwich ELISA Assay

Since dye-spectrometric methods have a limitation of sensitivity and specificity, direct measurements of specific GAGs, KS and HS, have been developed.

#### 2.2.1. Keratan Sulfate

The excessive accumulation of KS in cartilage is known to cause severe skeletal dysplasia in MPS IVA, and, therefore, appropriate quantitative method to measure KS in the blood of these patients was required. In 1988, Thonar *et al.* [36] developed an inhibition ELISA to measure blood KS level in healthy controls, suggesting that blood KS is age-dependent; however, this monoclonal antibody assay for KS measurement involves multiple laborious steps, leading to the requirement of improvements in methodology. In 2005, we showed that blood KS is unexpectedly elevated in other types of MPS, suggesting that blood KS could be used for screening, prognosing, and monitoring not only MPS IV but also other types of MPS [28].

**Figure 1:**
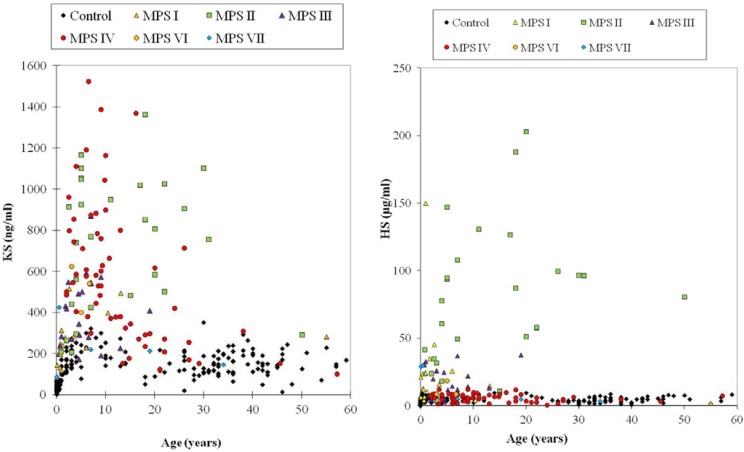
Plasma KS and HS levels determined by Sandwich ELISA assay. Left panel (plasma KS): The KS standards for ELISA calibration and the anti-KS monoclonal antibody (5-D-4) were obtained from Seikagaku (Tokyo, Japan). The ELISA procedure was described previously [28]. The absorbance was measured at 450 nm using a microplate spectrophotometer reference to 650 nm. The KS concentration was determined by applying the absorbance of each sample to the calibration curve [28]. Right panel (Plasma HS): All reagents described for HS-ELISA were provided from Seikagaku Co (Tokyo, Japan). Monoclonal antibodies against HS were used. The ELISA method was essentially the same as described for KS assay. The absorbance was measured at 450 nm (reference absorbance 630 nm) using a microplate spectrophotometer [29].

Plasma KS values more than 2SD above the mean of age-matched controls were found in MPS patients as follows (Figure 1; left panel); 16 out of 18 (88.8%) for MPS I, 27 out of 28 (96.4%) for MPS II, 16 of 20 (80%) for MPS III, 50 of 62 (80.6%) for MPS IV, 3 out of 3 for MPS VI, and 2 out of 5 (40%) for MPS VII. Thus, while over 80.0% patients with MPS IV had plasma KS levels more than 2SD above the mean of age-matched controls, for all other forms of MPS the proportion was even higher (more than 85%), indicating that elevated plasma KS is found in most MPS patients [28].

In urine, KS levels are not so markedly elevated in non-MPS IV patients. While 96.4% MPS IV patients had urine KS levels more than 2SD above the mean of age-matched controls, this value was only 22% for other MPS patients [28]. These findings demonstrate that there is a difference of magnitude of elevation in blood and urine KS between MPS IV patients and other MPS patients [28].

#### 2.2.2. Heparan Sulfate

In 2005, we established a sandwich ELISA method for HS measurement in MPS patients and showed that blood and urine HS levels are elevated in MPS I, II, III, VI, and VII [29].

HS levels in plasma showed that 18 of 22 (82.6%) attenuated and 65 of 67 (97%) severe patients with MPS I, II, III, and VII were more than 2SD above the mean of age-matched controls, indicating that severe patients had a higher frequency of elevated plasma HS.

Plasma HS values more than 2SD above the mean of age-matched controls are obtained as follows (Figure 1; right panel); 23 out of 33 (70%) for MPS I (2 of 5 attenuated; 22 of 29 severe), 29 of 33 (87.8%) for MPS II (8 of 11 attenuated; 21 of 22 severe), 23 of 30 (76.7%) for MPS III (23 of 28 severe; 0 of 2 attenuated), and 5 out of 9 (57.1%) for MPS VII patients (4 of 7 attenuated; 1 of 2 severe). Nine of 60 (15%) MPS IVA patients (1 of 13 attenuated; 8 out of 47 severe) had plasma HS values more than 2SD above the mean of age-matched controls. Plasma HS values in all five MPS VI patients were more than 2SD above the mean of controls [29].

Urine HS values in 13 of 27 (48.1%) attenuated patients and 82 of 89 (92.1%) severe with MPS I, II, III and VII were more than 2SD above the mean of age-matched controls, indicating a significant frequency of elevated urine HS in patients with severe forms of MPS. Twenty-nine of 75 (38.7%) MPS IVA patients (3 of 15 attenuated; 26 of 60 severe) had plasma HS values more than 2SD above the mean of controls. Three of seven MPS VI patients showed urine HS levels more that 2SD above the mean of controls [29].

Overall, most MPS patients who lacked enzymes required for catabolism of KS or HS had an elevation of KS or HS in blood and urine, respectively; however, many of these patients also had a secondary elevation of HS or KS despite having normal levels of enzymes requires to digest these particular GAGs. Thus, measurement of both KS and HS in blood and urine are likely to provide useful biomarkers to assess clinical severity at an early stage, and to monitor therapeutic efficacy for multiple forms of MPS [28,29,37]. A potential mechanism for these secondary elevations is discussed later in this review (see Figure 5).

## 3. History of GAG Assay by Tandem Mass Spectrometry (MS/MS)

Initial MS methods for GAG assays were developed to detect heparin oligosaccharide mixtures with electrospray mass spectrometry (EMS) [38] and disaccharides (KS, DS or CS) with high-performance liquid chromatography/turbo-ionspray tandem mass spectrometry tandem (LC-MS/MS) [39,40,41]. These methods are sensitive and accurate but its application is costly with some complicated procedures. Ramsay *et al.* developed a method for the derivatization and quantification of sulfated N-acetylhexosamine-containing mono- and di-saccharides from patient samples by electrospray ionization tandem mass spectrometry (ESI-MS/MS) [42,43]. Urine from most MPS types had significant increases in mono- and di-sulfated N-acetylhexosamines and mono-sulfated N-acetylhexosamine-uronic acid disaccharides. Analysis of plasma and DBS from patients with MPS indicated elevations of mono-sulfated N-acetylhexosamines but less than that seen in urine. This direct quantification of GAGs by mass spectrometry contained complex heterogeneous molecules with a variety of oligosaccharides.

**Figure 2:**
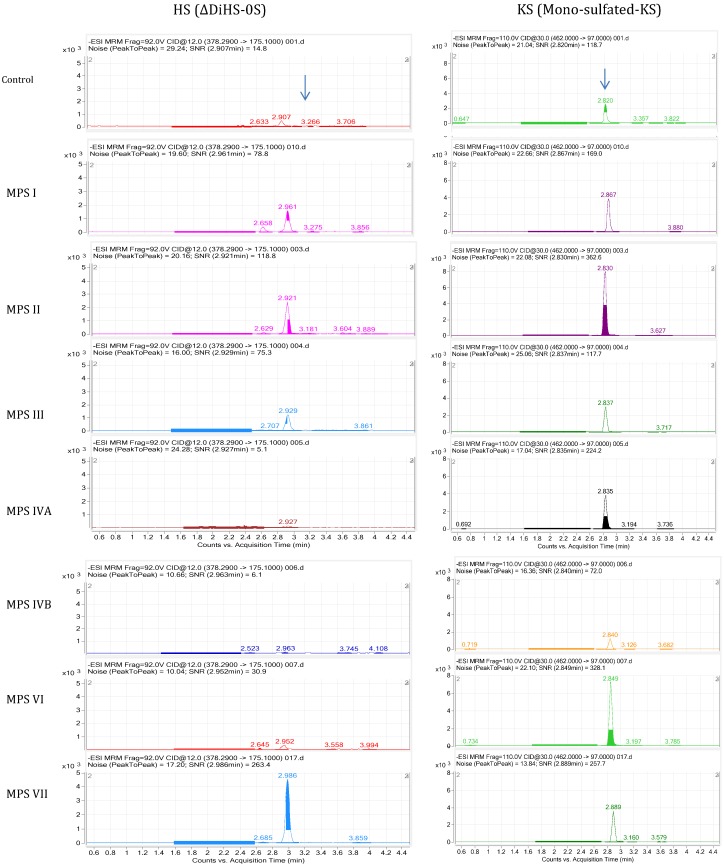
MRM of LC-MS/MS for plasma or serum samples in patients with MPS I-VII. ΔDiHS-0S (**left**) and mono-sulfated KS (**right**) are described.

The major GAGs that accumulate in MPS patients are co-polymers that contain analogous repeating units (glucurono-, or iduronosulfo-N-acetyl-glucosamine or galactosamine). These polymers are sensitive to chemical and enzymatic degradation (methanolysis and hydrolysis) to produce simple disaccharide molecules containing the repeating subunits. The sulfate groups are quickly cleaved, and the remaining acidic polysaccharides are hydrolyzed or methanolyzed at certain glycosidic linkages and separate to stable disaccharides as the end products. In 2007, we developed a highly sensitive, specific, accurate, and low-cost strategy. Disaccharides derived from DS, HS, and KS are produced in blood and urine specimens by hydrolysis, and then all products analyzed by a single LC-MS/MS method [44,45,46]. Other groups have subsequently followed to analyze the disaccharides with similar methods [47,48]. A significant advantage of this approach is that multiple disaccharides are measured from a single sample, and that all types of MPS can be detected. Blood, urine, or other biological samples are purified by filtration and the GAGs are digested with chondroitinase B, heparitinase, and keratanase II to yield disaccharides of DS, HS, and KS. After digestion, the samples are loaded into the LC-MS/MS for quantitation, and the results are compared with control samples (Figure 2). The LC-MS/MS method for analysis of disaccharides not only shows sensitivity and specificity for detecting all subtypes of MPS but also can monitor therapeutic efficacy in MPS patients and animal models [27,28,29,30,31,32,33,34,35,36,37,38,39,40,41,42,43,44,45]. This method has an advantage of being both GAG-specific and quantitative.

In 2011, Auray-Blais *et al.* described that methanolysis can be used to prepare disaccharides derived from HS and DS for LC-MS/MS analysis in urine of MPS patients [49,50]. Methanolysis has not yet been used to measure KS or blood samples. In 2012, Lawrence *et al.* showed that another new method with enzyme digestion can detect DS and HS by analysis of non-reducing ends of urinary GAGs by LC-MS/MS. Currently, this method does not provide a measure of non-reducing ends for KS [51]. Overall, establishment of methods to measure disaccharides makes it feasible to interpret individual GAG values, leading to feasible and accurate diagnosis, prognosis, and monitoring therapies for MPS.

Thus, several groups have contributed to methods that can assay disaccharides derived from CS, DS, HS, and KS in blood and/or urine samples by using LC-MS/MS for MPS.

The LC-MS/MS method for disaccharides not only shows sensitivity and specificity for detecting all subtypes of MPS but defines the clinical severity and monitor therapeutic efficacy in MPS patients and animal models [46,47,48,52,53,54,55,56,57]. The main drawback of the LC step is that the process is time consuming, limiting its utility for the screening of large numbers of samples.

Another unique method to measure the disaccharides of a specific GAG was developed by using RapidFire high-throughput mass spectrometry (HT-MS/MS). Samples are absorbed to a matrix to concentrate and desalt, and then eluted directly into the MS/MS without chromatographic separation. Each sample is processed in less than ten seconds, yielding much faster throughput than conventional LC-MS/MS based methods. A single 384 well plate can be read in ~45 min, indicating that this HT-MS/MS system can analyze over one million samples annually. The HT-MS/MS has been shown to provide sensitivity and specificity equivalent to standard MS/MS read-outs. The speed and efficiency of the HT-MS/MS can allow for experiments that would otherwise be deemed “untenable” under normal circumstances. The HT system has been validated as suitable for many drug discoveries [58,59,60,61,62,63,64,65] and ADME (Absorption, Distribution, Metabolism and Excretion) based applications [66]. The main drawback of this method is that disaccharides with identical molecular weights cannot be distinguished.

Newborn screening (NBS) is recognized as an essential, preventive public health program for early identification of diseases that can improve long-term health outcomes. Tandem mass spectrometry (MS/MS) has evolved as a powerful tool to detect small compounds because the detection is based on the mass of the parent compound and a specific fragment(s) of the compound.

## 4. MS/MS Method for Disaccharides

### 4.1. Standards and Enzymes

To digest “polymer” DS, HS, and KS to disaccharides, chondroitinase B, heparitinase, and keratanase II were provided from Seikagaku Co. (Tokyo, Japan). Heparitinase digests HS to yield the disaccharides ΔDiHS-0S, ΔDiHS-NS, and ΔDiHS-6S; chondroitinase B digests DS to yield ΔDi-4S; and keratanase II digests KS to yield Galβ1→4GlcNAc (6S) and Gal (6S) β1→4GlcNAc (6S) (mono- and di-sulfated KS disaccharides).

Chondrosine was used as an internal standard (IS). The following standards were used to make standard curves of each specific disaccharide; ΔDi-6S (C6S) [2-acetamido-2-deoxy-4-O-(4-deoxy-a-L-threo-hex-4-enopyranosyluronic acid)-6-O-sulfo-D-glucose], ΔDi-4S (DS) [2-acetamido-2-deoxy-4-O-(4-deoxy-L-threo-hex-4-enopyranosyluronic acid)-4-O-sulfo-D-glucose], ΔDiHS-0S [2-acetamido-2-deoxy-4-O-(4-deoxy-a-L-threo-hex-4-enopyranosyluronic acid)-D-glucose]; ΔDiHS-NS [2-deoxy-2-sulfamino-4-O-(4-deoxy-a-L-threo-hex-4-enopyranosyluronic acid)-D-glucose]; ΔDiHS-6S (HS) [2-acetamido-2-deoxy-4-O-(4-deoxy-a-L-threohex-4-enopyranosyluronic acid)-6-O-sulfo-D-glucose]; mono-sulfated KS-Galβ1-4GlcNAc(6S); di-sulfated KS - Gal(6S)β1-4GlcNAc(6S). All standards were provided by Seikagaku Corporation (Tokyo, Japan).

### 4.2. Sample Preparation

Plasma/serum, urine, DBS, and standards were prepared as follows. Ten μL of each plasma/serum and urine sample and 90 μL of 50 mM Tris–hydrochloric acid buffer (pH 7.0) were placed in wells of AcroPrep^™^ Advance 96-Well Filter Plates that have Ultrafiltration Omega 10 K membrane filters (PALL corporation, NY, USA). The filter plates were placed on the receiver and centrifuged at 2000 g for 15 min to remove free disaccharides. The membrane plates were transferred to a fresh receiver plate. Standards were added to unused wells of the filter plate.

Ten μL of IS solution (5 μg/mL), 20 μL of 50 mM Tris-HCl buffer, and 10 μL of chondroitinase B, heparitinase, and keratanase II, respectively (each 2 mU/10 μL of 50 mM Tris-HCl buffer), were added onto each filter. The plate was incubated at 37 °C for 5 h and centrifuged at 2000 g for 15 min. The processed samples were injected to LC-MS/MS or HT-MS/MS or stocked at −20 °C until use.

### 4.3. LC-MS/MS

We describe here the current LC-MS/MS method using enzyme digestion based on that originally developed by Oguma *et al.* [30,44,45]. We used two different LC-MS/MS instruments to quantify disaccharides; an HP1100 LC system (Agilent Technologies, Palo Alto, CA, USA) with API-4000 (AB Sciex, Foster City, CA, USA) and a 1260 infinity LC system with 6460 Triple Quad (Agilent Technologies, Palo Alto, CA, USA) [30]. A Hypercarb column (2.0 mm i.d. 50 mm, 5 µm, Thermo Electron, USA) was used with both chromatographic systems. The mobile phase was a gradient elution from 0.025% ammonia to 90% acetonitrile in 0.025% ammonia. The mass spectrometer was operated in the negative ion detection mode with thermal gradient focusing electrospray ionization. Specific precursor ion and product ion were used to quantify each disaccharide [32,67,68]. A *m/z* 354.29 precursor ion and *m/z* 193.1 product ion was used to detect the IS (chondrosine). The concentration of each disaccharide was calculated using Quantitative Analysis software.

### 4.4. High-Throughput Tandem Mass Spectrometry (HT-MS/MS)

Sample plates containing the disaccharides were centrifuged to collect all samples to the bottom of the plate, and applied to a RapidFire 200 (Agilent Technologies, Inc: Boston, MA, USA), followed by MS/MS in an API 4000 (ABSciex, Framingham, MA, USA) [30]. In the multiple reaction monitoring (MRM) mode, the mass spectrometer detected ions by monitoring the decay of specific precursor ion to the product ion for each disaccharide. The decay of the *m/z* 354.29 precursor ion to the *m/z* 193.1 product ion was used for IS. The files from HT-MS/MS were merged to identify peaks and analyzed to determine the area under the curve (AUC). The levels of each disaccharide were measured and averaged.

### 4.5. Disaccharide Determination Derived from GAGs

#### 4.5.1. LC-MS/MS

Using purified preparations of ΔDiHS-0S, ΔDiHS-NS, and ΔDi-4S, we optimized MRMs for each individual sugar and obtained single peaks corresponding to the pure compound. All disaccharides are eluted in less than 3 min. The detection method for mono-sulfated Galβ1-4GlcNAc(6S) (KS1) also detected some Gal(6S) β1-GlcNAc(6S) (KS2) at the same MRM but the two forms were clearly separated by chromatography. MRMs for ΔDiHS-0S and mono-sulfated KS patterns in MPS I-MPS VII are shown in Figure 2.

#### 4.5.2. HT-MS/MS

**Figure 3:**
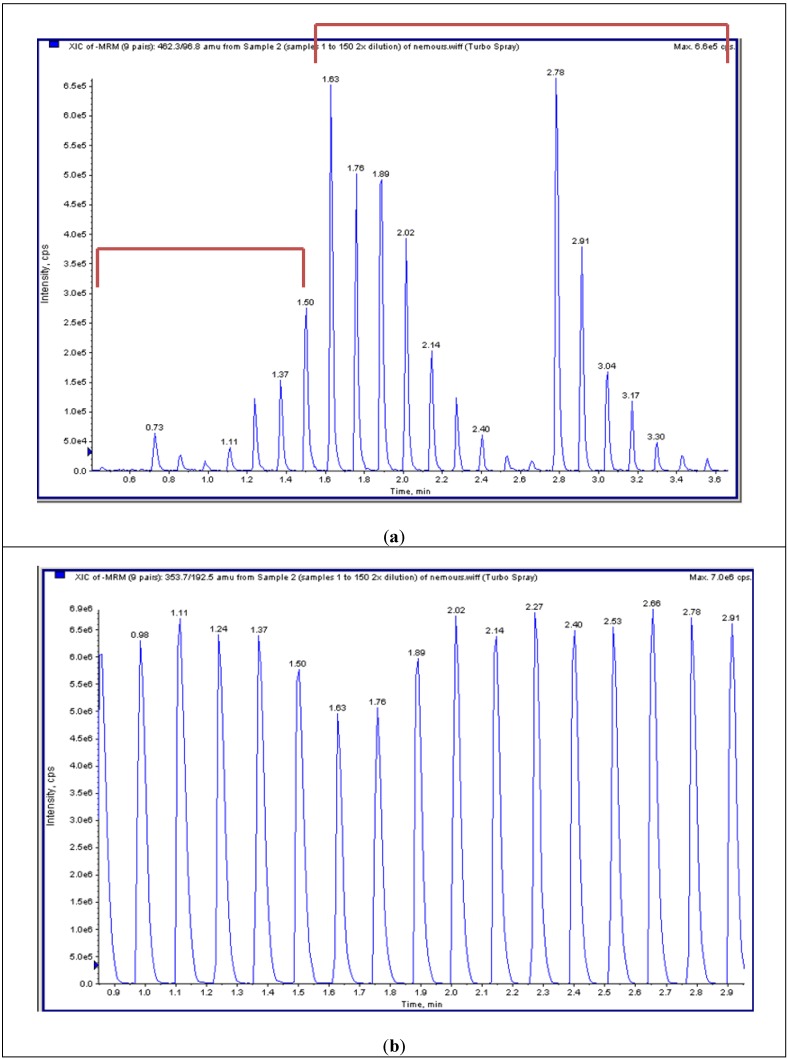
Multiple reaction monitoring (MRM) of HT-MS/MS. (**a**) Multiple injections of mono-sulfated KS with a series of dilutions in duplicate shows seven gradient peaks per a set of dilutions (as well as other surrounding samples). (**b**) Multiple injections of chondosine (Q1; 353.7, Q3; 192.5) with the same concentration shows 8 peaks per min.

The HT system does not have a chromatographic separation step but rather samples are first bound to solid phase matrix for desalting and concentration and then eluted directly into an MS/MS system. HT-MS/MS cannot distinguish disaccharides with identical molecular weights that give the same MRMs; however, this MS/MS can distinguish and quantify combinations of different disaccharides with a higher throughput (for example, see mono-sulfated KS in Figure 3).

### 4.6. Analysis of Plasma and Serum Samples in MPS Patients by LC-MS/MS

Each type of MPS has a different pattern of elevated GAG levels in the blood. We described the concentrations of DS-, HS-, and KS-derived disaccharides and their composition ratios in human control and MPS patients [44,45,46].

#### 4.6.1. Keratan Sulfate

**Figure 4:**
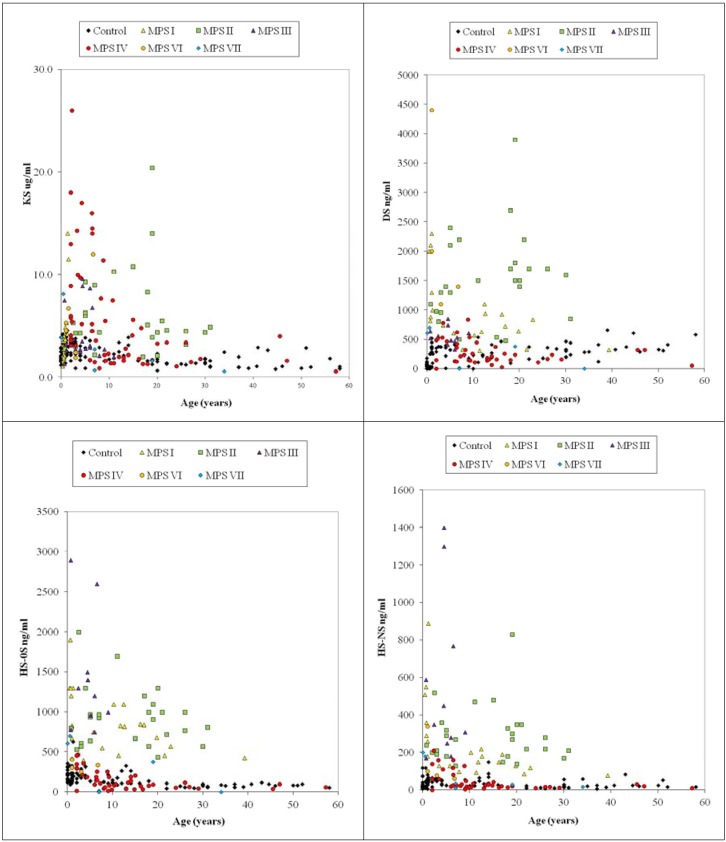
Plasma KS, DS, and HS (ΔDiHS-0S and ΔDiHS-NS) levels by LC-MS/MS.

Blood KS concentrations were found to vary with age as shown by ELISA (Figure 1). In healthy control newborns, blood KS concentration was under 2.0 μg/mL, and it rose, reaching a peak between 0 and 2 years of age, and the concentrations stayed relatively constant until the individual reached 15 years of age. After 15 years, blood KS concentrations decreased gradually and stabilized thereafter. When control subjects and MPS IVA patients in each age range were compared, the difference in KS levels between MPS IVA patients and the age-matched controls was greater at younger ages. It is noteworthy that KS level in patients with MPS IVA reduced with age and is almost normalized by the age of 15 years old. Blood KS levels in severe MPS IVA were higher than in the attenuated form. This finding indicates that KS level could be associated with clinical severity. The level of plasma KS was also compared between each type of MPS and age-matched controls. Plasma KS levels in 4 out of 31 (12.9%) MPS I patients were more than 2SD above the mean of age-matched controls (Figure 4). Patients with MPS II had the highest mean KS in their blood among all types of MPS patients except MPS IV. Twenty-two out of 28 (78.6%) of MPS II patients had plasma KS values more than 2SD above the mean of age-matched controls (Figure 4). Plasma KS values in 5 of 19 (26.3%) MPS III patients, 3 of 6 (50%) MPS VI patients, and 1 of 6 MPS VII patients were more than 2SD above the mean of age-matched controls (Figure 4).

The accumulation of undegraded KS leads to damage of cartilage cells, causing systemic skeletal dysplasia in patients with MPS IVA. KS, which contributes over 25% of the cartilage GAGs in adults, is one of the most important components in bone. When cartilage proteoglycans, such as KS, are not degraded properly, they are stored mainly in chondrocytes, where KS is synthesized. Pathological examinations of the bone and cartilage cells are useful for the diagnosis of MPS IVA; however, it is not practicable to obtain biopsy samples from every MPS IVA patient.

Levels of elevation of KS detected by LC/MS/MS in plasma for MPS I, II, VI and VII are not as great as seen using ELISA methods and may reflect differences in the two assays or differences in the patient populations studied. The monoclonal antibody used for sandwich ELISA (Seikagaku, Tokyo, Japan) is specific for Galβ1(6S)→4GlcNAc(6S); *i.e.*, both galactose and N-acetyl-glucosamine have to be sulfated [69,70]. Therefore, this analytical method does not provide quantification of total KS. Notably, total KS measured by LC/MS/MS was 10–100 times higher than measured by the sandwich ELISA method and the correlation between plasma KS measurements assayed by both methods for MPS IVA patients was weak. The ELISA method could also underestimate total and relative levels of KS due to poor quantitation of different degrees of KS polymerization. The LC/MS/MS method is also more sensitive than the ELISA method. While the ELISA method used in the study could not detect below 2.5 ng/mL of KS, LC/MS/MS could detect 0.2 ng/mL of KS. Consequently, in rodents that synthesize much less KS, blood levels are measurable by LC/MS/MS but not by ELISA. Although ELISA methods do not require expensive equipment, LC/MS/MS methods are less expensive per sample and appear to be more sensitive and accurate.

#### 4.6.2. Dermatan Sulfate and Heparan Sulfate

Plasma DS: All 4 MPS VI patients had a significant elevation of plasma DS, compared with the controls. MPS I (18/22, 81.8%), MPS II (26/27, 96.3%), MPS III (5/11, 45.5%), MPS VII (2/7, 28.6%) and ML II (3/7, 42.9%) patients also had a significant elevation of DS. These findings suggest that the measurement of DS levels by LC/MS/MS is applicable to the screening for MPS I, II, III and VI patients (Figure 4) [46].

Urine DS: All MPS VI patients (n = 3) had a marked elevation of urine DS, compared with the controls. All MPS I (n = 17) and MPS II (n = 19) patients also had a significant elevation. Two of three MPS III, two of five MPS IVA, and one of two MPS VII patients showed a significant elevation [46].

Thus, the secondary elevation of blood and/or urine DS was found in some of MPS III and IVA patients, although these patients should have normal levels of the enzymes needed to digest DS.

Plasma HS: All MPS I, II, and III patients (60 out of 60 patients) had a significant elevation of plasma ΔDiHS-0S and ΔDiHS-NS, compared with the controls (Figure 4): The MPS III patient population included five IIIA patients, four IIIB patients and two IIIC patients. Two out of 6 MPS VII patients also had a significant elevation of HS [46].

Urine HS: Most MPS I, II, III and VII patients (36/39; 92.3%) had a significant elevation of urine HS (ΔDiHS-0S and ΔDiHS-NS), compared with age-matched controls. Two out of five MPS IVA and two out of three MPS VI patients also had a significant elevation [46].

Thus, the secondary elevation of blood and/or urine HS was observed in some of MPS IVA and VI patients (Figure 4), although these patients should have normal levels of the enzymes needed to digest HS.

#### 4.6.3. Composition of DS and HS in Blood

The compositional ratio of ΔDiHS-0S, ΔDiHS-NS and ΔDiDS in total DS and HS derived from blood samples of MPS patients was compared. The ratio of the compositions was expected to be reflected by deficiency of each enzyme. For the normal control individuals, the mean ratio of ΔDiHS-0S, ΔDiHS-NS, and ΔDiDS in total DS and HS was 40.4%, 7.7%, and 51.9%, respectively. The proportion of ΔDiDS was significantly higher in MPS VI patients compared to that in normal controls (mean; 80.6% *vs.* 51.9%). The proportion of ΔDiHS-0S was significantly higher in MPS III and VII patients compared to that in normal controls (mean; 56.4% *vs.* 40.4%; 65.1% *vs.* 40.4%), suggesting that ΔDiHS-0S is dominant in MPS III and VII. The proportion of ΔDiHS-NS was also significantly higher in MPS III patients compared to that in normal controls (mean; 19.7% *vs.* 7.7%). Other types of MPS did not provide any significant difference in ratios of DS and HS.

### 4.7. Analysis of Plasma and Serum Samples in MPS Patients by LC-MS/MS

We analyzed plasma and serum samples for DiHS-NS and DiHS-0S in control subjects by both LC-MS/MS and HT-MS/MS. Initial analyses of ΔDiHS-NS and ΔDiHS-0S by LC-MS/MS and HT-MS/MS indicates that the results from each assay are comparable [30]. Furthermore, in a larger studyHS levels were measured in plasma/serum from both control subjects and patients with MPS II, III, and IV [71].

The results showed (1) that there was a strong correlation of signals for disaccharides derived from ΔDiHS-NS and ΔDiHS-0S between conventional LC-MS/MS and HT-MS/MS, (2) that blood levels of ΔDiHS-NS, ΔDiHS-0S were significantly elevated in patients with MPS II and III, but not in patients with MPS IVA, (3) that the level of HS in patients with a severe form of MPS II was higher than that in an attenuated form, and (4) that reduction of blood HS level was observed in MPS II patients treated with enzyme replacement therapy or hematopoietic stem cell transplantation.

In conclusion, HT-MS/MS provides much higher throughput than LC-MS/MS-based methods with similar sensitivity and specificity in an HS assay, indicating that this method is feasible for diagnosis, monitoring, and screening of MPS. DS and KS levels are now under investigation. Once we have established all GAG assays by HT-MS/MS, we will be able to determine the relative advantages and disadvantages of LC-MS/MS and HT-MS/MS.

### 4.8. Newborn MPS

It is of great interest to know when GAGs begin to accumulate in tissues of the affected patients to apply GAG assays to newborn screening and to understand the correlation between GAG accumulation and the appearance of clinical signs and symptoms. Whether the elevation of GAGs is detectable in newborns with MPS was not known until recently. To evaluate if the LC-MS/MS method distinguishes MPS newborns from healthy control newborns, we measured DS and HS levels in DBS samples from six newborns with MPS (four MPS I, one MPS II, and one MPS VII) and compared them with control newborns. All six cases had significant elevations of DS and HS compared with the values of normal control newborns [67].

Similarly, Ruijter *et al.* assayed DS and HS levels from newborn DBS of 11 MPS I, 1 MPS II, and 6 MPS III patients, with phenotypes ranging from severe to relatively attenuated forms. The levels of DS and HS derived disaccharides in these DBS were compared with levels in DBS of newborn controls. The levels of DS and HS derived disaccharides were elevated in all newborn DBS of MPS I, II, and III patients when compared with controls [48].

Based on these results, we propose that DS and HS are a useful diagnostic and screening marker for MPS I, II, III, VI, and VII individuals.

## 5. Secondary Elevation of KS

There is a certain correlation between types of MPS and accumulated GAG(s) based upon the degradative enzyme affected (2). Elevation of KS level in blood and urine specimens was deemed as a hallmark of MPS IV since the deficient enzyme is directly engaged in catabolism of KS; however, MPS disorders other than MPS IV are also associated with elevated KS levels in blood and urine, in addition to the GAG accumulating due to the primary enzyme defect [28,31]. In particular, KS elevation in blood is more prominent than that in urine and is as high as the levels seen in patients with MPS IVA for many MPS sub-types. The elevation of urine KS in other types of MPS was not previously recognized due to the low sensitivity of conventional thin-layer chromatography (TLC) methods compared with the LC-MS/MS or ELISA methods described here.

The majority of KS is synthesized in cartilage tissue, and the metabolized KS is secreted into the blood and excreted in urine. KS level in the normal population reaches a peak during the growth spurt and gradually decreases with age when the growth plate is being closed. MPS IV is caused by deficiency of the enzyme directly involved in KS degradation pathway, leading to accumulation of undegraded KS, mainly in cartilage. The resulting accumulation of undegraded KS damages the chondrocytes and their ECM. The structure of PGs and collagen in cartilage become abnormal, resulting in apoptosis of chondrocytes and the release of KS from the cells to the body fluids. The extent of elevated KS in blood and urine in patients with MPS IVA correlates positively with clinical severity and its prognosis at a progressive stage [28,31,54]; however, KS level in patients decreases with age after 10 years of age, earlier than that in the normal population since the main synthetic site of KS in cartilage is destroyed and lost. Blood KS in MPS IVA is becomes similar to that seen in controls after patients become teenagers [28,31,54].

It is of importance to understand why KS is elevated in other types of MPS because the current understanding of the pathway of KS catabolism cannot account for this event.

**Figure 5:**
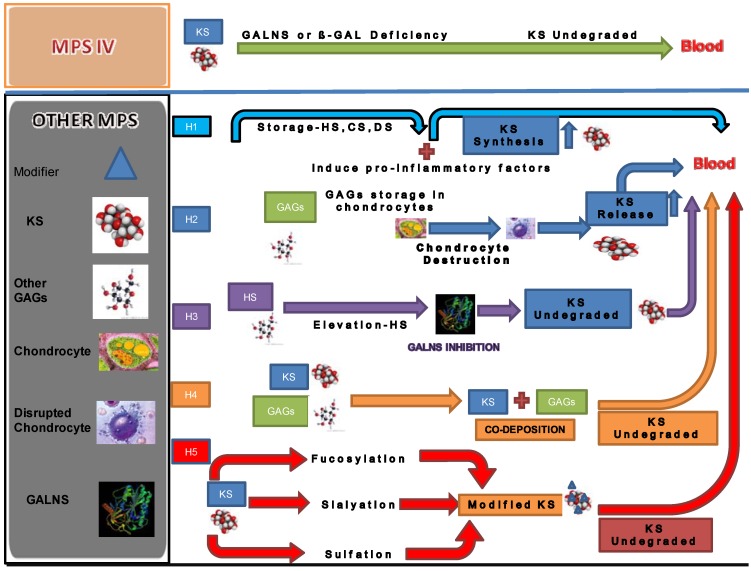
Hypotheses (H) of secondary elevation of blood KS in other MPS. H1; Accumulation of GAGs and pro-inflammatory factors induces KS synthesis. H2; GAG storage in chondrocyte leads to cell death and release of KS. H3; Accumulated HS leads to inhibition of GALNS activity. H4; Other GAGs co-deposited with KS masks the access of degradative enzymes for KS and undegraded KS is too large for filtration in kidney. H5; Fucosylation, sialyation, or sulfation modification of KS leads to resisting degradation.

Several hypotheses have been proposed to explain the elevation of blood KS in patients with other types of MPS as follows (Figure 5):

(1) The synthesis of KS is stimulated by storage of other GAGs. Accumulation of GAGs can induce pro-inflammatory factors such as IL-1β, 6, and 10 and TNF-α [14]. Accumulated GAGs and/or pro-inflammatory factors promote the synthesis of KS secondarily.
(2) The elevation of KS is a secondary consequence caused by skeletal dysplasia. Accumulation of other GAGs could cause inflammation and thereby damage cartilage and its ECM leading to increased secretion of KS into the circulation. Degradation of PGs occurs early in joint damage. The fragments of PGs are released into the synovial fluid and subsequently the blood [72,73]. This hypothesis is supported by the fact that KS levels in patients with MPS I, II, and VI are more elevated, compared with that in MPS III that results in less marked skeletal dysplasia. KS elevation is more prominent in patients with a severe form of MPS II than in an attenuated phenotype [28,31]. An MPS VII mouse model has a severe skeletal abnormality and blood KS level is elevated more than other mouse models of MPS that have less marked skeletal abnormalities [74]. Elevated levels of KS were also seen in an MPS I mouse model but was limited in MPS IIIA and MPS IVA mice. These findings suggest that blood KS elevation is caused by release of KS from chondrocytes damaged due accumulation of other GAGs and subsequent inflammation. Paradoxically, in the MPS IVA mouse model a severe bone dysplasia is not seen and KS elevation in blood is limited despite the absence of functional GALNS enzyme. Thus, although KS would be expected to accumulate in cartilage of MPS IV mice, it is primarily the severity of bone dysplasia that correlates with levels of KS measured in blood.
(3) GALNS activity is inhibited by HS that accumulates in patients with MPS. HS is known to directly inhibit GALNS enzyme activity in vitro. MPS IIIA, I and VII mice have increasing levels of HS and also increasing levels of serum KS, indicating a correlation between HS inhibition of GALNS and increased KS [74]. Patients with a severe form of MPS II have a high level of HS and a more prominent increase of KS at a young age. These findings support the hypothesis that inhibition of the GALNS enzyme by elevated HS causes a secondary elevation of KS levels in MPS I, II, III, and VII patients.
(4) Polymer KS interacts and co-deposits with other accumulated GAGs. Co-deposition with other GAGs hinders the interaction between KS and enzymes that catabolize KS. Elevation of KS was less striking in urine than in blood for MPS other than MPS IV. The lower elevation of urine KS in patients with other forms of MPS could be explained by aggregation of KS with other GAGs or unknown factors in the bloodstream. The aggregates may be too large to be cleared into the urine. In this scenario, undegraded KS not filtered out by the kidney remain retained in the blood.
(5) Changes in the pattern of fucosylation, sialylation, and sulfation on KS secondary to other GAG accumulation makes KS resistant to degradation. KS derived from articular cartilage contains sialic acid and fucose. This hypothesis is supported by the fact that sialic acid residues, present as chain caps, and fucose residues inhibit degradation of KS molecules [75].

One or a combination of these factors may contribute to secondary elevation of KS. Further study will elucidate the mechanism.

Most MPS patients have severe bone dysplasia. Therefore, elevated KS in the blood of other MPS patients could relate to underlying bone disease, especially cartilage tissues. Blood KS provides more specificity for the bone pathology of MPS disease than the originally stored substrates HS and/or DS. It would be useful to investigate the KS levels in the blood as a biomarker of other MPS. It is noteworthy to know whether KS concentrations are elevated in selected newborn babies with MPS from the viewpoint of early detection. Although MPS are progressive disorders that often take years to present clinically, there is considerable evidence from both in humans [76,77,78] and in animal models [79] that biochemical storage commences in the fetus.

## 6. Conclusions

We conclude that LC-MS/MS and HT-MS/MS provide a comparable sensitivity and accuracy for simultaneous measurement of three GAGs (DS, HS, and KS) and could be used for prognosis, diagnosis, monitoring, and screening. ELISA is another feasible and reproducible method although the variety of GAGs measurable is limited. Advantage of use of LC-MS/MS is that GAGs with the same molecular weight can be separated leading to accuracy and specificity of the differential diagnosis. HT-MS/MS provides a higher throughput and has the potential of being applied to screening for most inherited metabolic disorders, although this system cannot recognize the molecules with the same molecular weight separately. Secondary elevation of blood KS in other types of MPS in addition to MPS IV remains unsolved, but it should be valuable for diagnosis, monitoring, and screening for MPS related with skeletal dysplasia. Sulfation patterns of each specific GAG contribute to not only critical biological roles but also the differential diagnosis of MPS type.
